# Homogenization Method for Modeling and Analysis of the Honeycomb Structure—Simulation Study and Validation

**DOI:** 10.3390/ma18163884

**Published:** 2025-08-19

**Authors:** Łukasz Michalski, Tomasz Kubiak, Luigi De Mercato

**Affiliations:** 1 Faculty of Mechanical Engineering, Lodz University of Technology, ul. Stefanowskiego 1-15, 90-924 Lodz, Poland; tomasz.kubiak@p.lodz.pl; 2 Hitachi Energy, Brown-Boveri Strasse 5, 8050 Zurich, Switzerland; luigi.de-mercato@hitachienergy.com

**Keywords:** mathematical modeling, homogenization method, representative volume element (RVE), honeycomb panels, numerical simulation

## Abstract

This study presents a comprehensive approach to modeling honeycomb structures using the homogenization method, utilizing a Representative Volume Element (RVE) to derive equivalent orthotropic mechanical properties of the honeycomb structure. Three finite element models (two-dimensional and three-dimensional) were examined, considering the decreasing complexity and computational effort associated with each modeling approach. The outcomes of the analysis for each modeling approach were reported, with attention to boundary condition application and numerical singularities. Validation through four-point bending tests and an analytical approach confirmed the model’s ability to replicate the mechanical behavior of the panel. The obtained results have shown perfect agreement between the results of the numerical test employing the proposed model and the experimental test results of real structures. It was found that the proposed simplified numerical model allows for a reduction in the calculation time of c.a. 54%. Additionally, some disadvantages of using procedures included in commercial software such as a black box have been shown.

## 1. Introduction

The honeycomb structure is known for its exceptional mechanical properties and lightweight characteristics. It has gained significant attention in various engineering applications, and it is widely used in fields such as aerospace [1], automotive [2], marine [3], construction [4], furniture [5], and general industry [6].

Accurate modeling of the mechanical behavior of honeycomb panels is important when designing and optimizing structures utilizing them. In recent years, numerous research studies, such as the work of Dung et al. [7], Ali et al. [8], or Buitrago et al. [9], have focused on developing numerical models to capture the mechanical response of honeycomb structures under different loading conditions.

This paper aims to present a comprehensive modeling approach for honeycomb structures using the homogenization method [7,10]. Homogenization is a technique that enables the effective representation of complex microstructures by averaging their mechanical properties at a macroscopic scale. By applying homogenization, it becomes possible to obtain an equivalent model that accurately represents the behavior of honeycomb structure.

The main focus of the described work was on developing an efficient finite element model for honeycomb core composite panels, enabling simple and fast simulation, facilitating the selection of panels with appropriate mechanical properties for specific applications. To simplify the analysis of complex structures like honeycomb cores, the approach of homogenization using a Representative Volume Element (RVE) is utilized. Three finite element models (two-dimensional and three-dimensional) were examined in order considering the decreasing complexity and computational effort associated with each modeling approach. The outcomes of the analysis for each modeling approach were reported. Particular attention was given to the accurate application of boundary conditions and to the identification and assessment of numerical singularities.

To validate the proposed modeling approach, reference was made to experimental results and analytical solutions from previous research studies of the author and G. Moroni performed on behalf of the Hitachi Energy company (former ABB). The aim was to demonstrate the capability of the proposed homogenization-based modeling approach in effective representation of the mechanical behavior of honeycomb structures.

Several scientific papers related to honeycomb structure modeling served as valuable references for this study. The work of Carrera et al. [11], additionally to the already mentioned studies conducted by Ali et al. [8] and Buitrago et al. [9], contributed to the understanding of numerical modeling methods of honeycomb structures.

This paper presents different modeling approaches to honeycomb structures and tries to find an optimal way by means of a homogenization method. The simulation study conducted aims to demonstrate the capability of the proposed approach to accurately predict the mechanical behavior of honeycomb structures under various loading conditions. By validating the modeling approach through comparisons with experimental results and analytical solutions from the relevant literature [12,13], this study contributes to the field of honeycomb structure modeling and provides insights for the design and optimization of such structures in engineering applications.

## 2. Materials and Methods

### 2.1. Honeycomb Panels

Composite structures might have mechanical properties that outperform that of the individual materials they are composed of. Better mechanical performance is usually provided with lower weight and often a more compact build compared to, e.g., standard construction steel. Multi-structural materials (honeycomb core panels, wave core panels, laminates, etc.) are commercially available and widely used these days.

**Figure 1:**
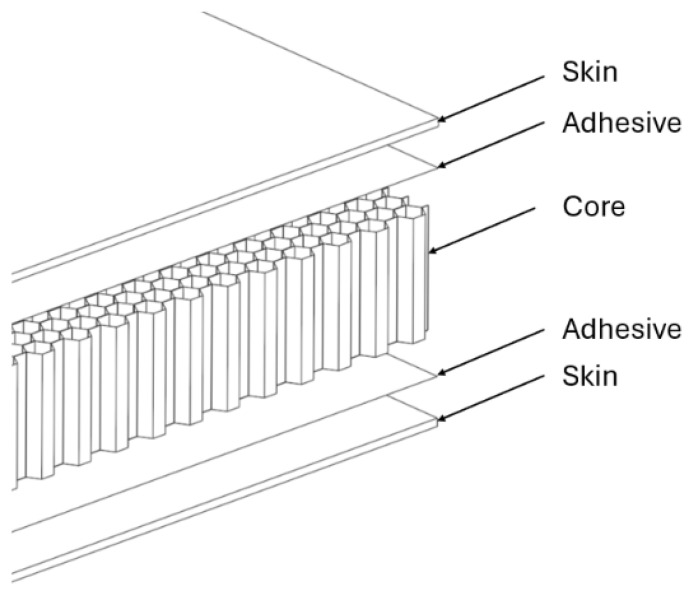
Basic structure of honeycomb composite panel.

The focus of this paper is placed on a honeycomb core sandwich panel, which is most often composed of two face sheets bonded to a honeycomb core by adhesive. An overview of the basic panel construction is shown in Figure 1.

Layered construction with a honeycomb-patterned core results in a high-strength, lightweight structure that reacts to bending moments, much like an I-beam. In principle, those are two plates separated by a stiff distancing structure. The highest mechanical properties of such a panel can be observed when loaded in bending. In such circumstances, high resistance to out-of-plane shear stresses is shown. Additionally, the honeycomb structures have a good level of energy absorption in case of impact load [14,15,16] and are characterized by good damping levels in case of harmonic load or vibration [17,18].

**Figure 2:**
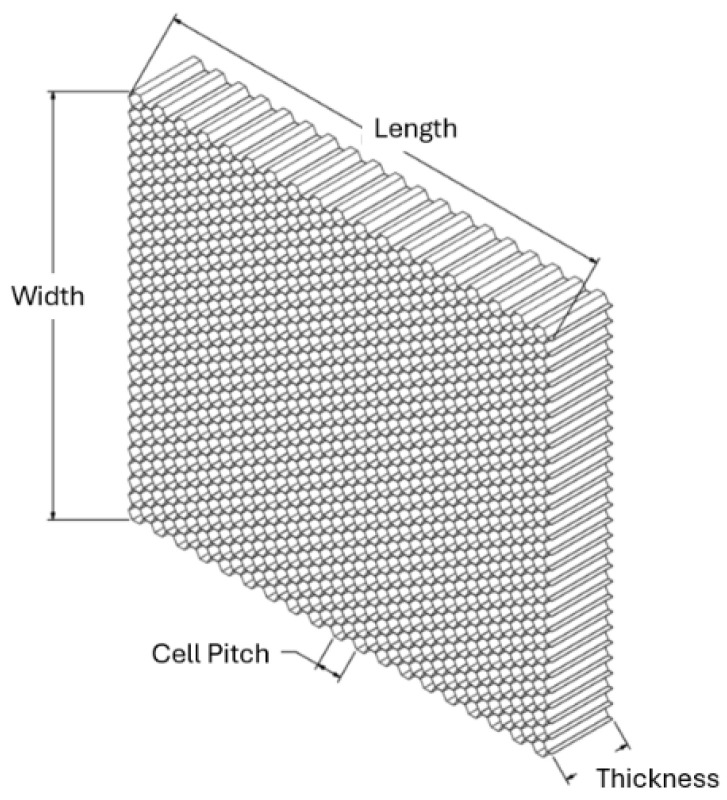
Honeycomb core geometry overview.

A honeycomb core structure can be seen in Figure 2. Properties can be customized by different core/overall sizing and material selection.

Key advantages include the following:

1. High flexural stiffness;
2. High strength-to-weight ratio;
3. Finished edges for mechanical or structural purposes (e.g., connection);
4. Multiple manufacturing options;
5. Good level of energy absorption in case of impact load;
6. Good damping level in case of harmonic load or vibration.

The regular geometry of honeycombs (periodical in each direction) enables extraction of mechanically equivalent properties.

### 2.2. Core Homogenization

Homogenization is a method used to analyze complex materials or structures by treating them as uniform structures. It involves averaging or smoothing out the properties of different components to create a simplified model. This helps to reduce computational complexity, especially in FEM, by reducing the number of elements, nodes, and consequently number of DoFs. Homogenization provides a simpler way to assess the behavior of the system. This method is widely used in various fields to study composite materials and other heterogeneous systems.

**Figure 3:**
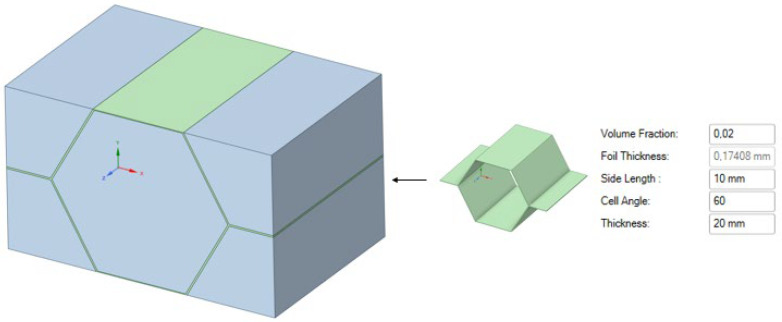
RVE (left side) of the honeycomb cell with given geometrical properties (right side).

Representative Volume Element (RVE), shown in Figure 3, is a small volume of the material that is still large enough to exhibit the correct macroscopic material properties. For a honeycomb-shaped core, this can be easily identified as one unit cell. In a periodic material, this unit cell repeats itself in all three coordinate directions. Thus, it contains all the information about the material, and it is sufficient to consider only the behavior of the single unit cell [19].

Homogenization is applied to the core of the sandwich panel, where complex shapes of ribbons which form the honeycomb are replaced with a homogeneous block (Figure 3) with orthotropic material properties, allowing it to retain its physical properties.

### 2.3. Theoretical Background

The mathematical model of the homogenization process for the honeycomb core presented in this chapter is largely based on the work of L. Dung et al. [7], S. Sorohan et al. [20], and E. Sather et al. [21]. The theoretical background presented below summarizes the necessary knowledge needed to understand the homogenization of the material process.

Equivalent mechanical properties of honeycombs can be obtained by using the stress–strain relationship [20], starting with the isotropic properties of the core material:

$\rho_{s}$—mass density,

$E_{s}$—Young’s modulus,

$\nu_{s}$—Poisson’s ratio,

$G_{s}$ = ½$E_{s}$/(1 − $\nu_{s}$)—Shear modulus,

and the known geometrical properties of the single honeycomb core cell being as follows:

b—height of the core (thickness of the core layer),

t—foil thickness,

h, l—dimensions of the cell walls,

θ—cell walls angle,

$L_{x}$, $L_{y}$—cell span 

**Figure 4:**
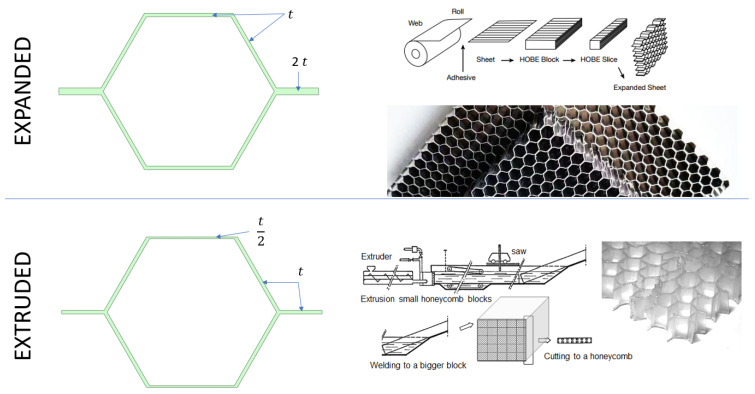
Expanded vs. extruded honeycomb core production method.

It is important to mention that there are two common cases for honeycomb cell modeling coming from two production methods largely used in the industry. Those two methods are expanded and extruded cores, and can be seen in Figure 4 below. It is mentioned here due to the difference in single cell geometry.

#### 2.3.1. Expanded Core

**Figure 5:**
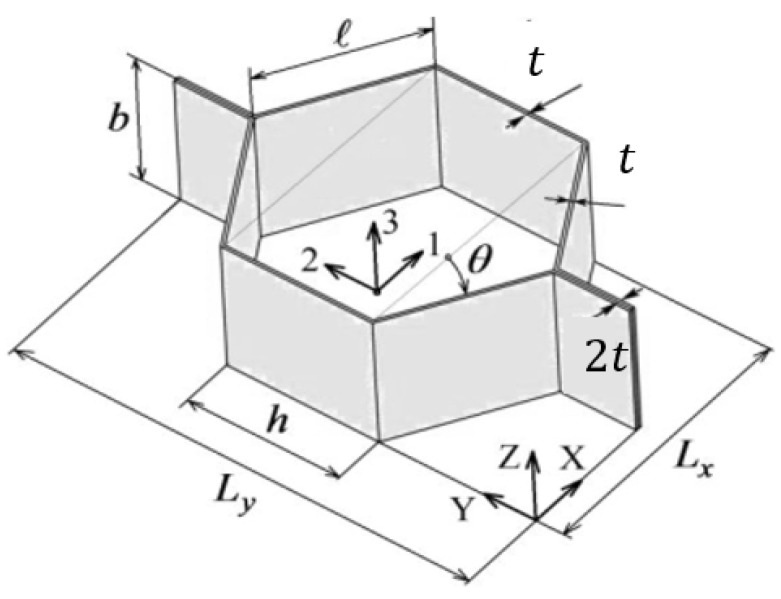
Honeycomb core cell with equation symbols listed for expanded core.

The section below focuses on describing mathematical equations for obtaining homogenized properties of cells produced using expanded core manufacturing processes. Figure 5 associates symbols which are later used in equations, to the geometrical properties of the core cell.

Equations to calculate the properties of the honeycomb core produced by expansion have been proposed by E. Sather et al. [21].

Relative density is calculated after equating the volume of the real honeycomb structure with the volume of the homogenized block:

(1) $$\frac{\rho }{\rho_{s}}=\frac{\frac{t}{l}\left(\frac{h}{l}+2\right)}{2\left(\frac{h}{l}+\sin\theta \right)\cos\theta }$$

Youngs’ moduli in X, Y, and Z directions are:

(2) $$E_{xx}=\frac{E_{c}{\left(\frac{t}{l}\right)}^{3}\cos\theta }{\left(1+\sin\theta \right)\sin^{2}\theta }\;;\;E_{yy}=\frac{E_{c}{\left(\frac{t}{l}\right)}^{3}\left(1+\sin\theta \right)}{\cos^{3}\theta \;}{;\;E}_{zz}=E_{c}\frac{\rho }{\rho_{c}}$$

where *E_c_* is elastic modulus of a cell wall.

Poissons’ ratios in all three planes:

(3) $$\nu_{xy}=\frac{{cos}^{2}\theta }{\left(1+\sin\theta \right)\sin\theta }\;;\;\nu_{xz}=\nu_{c}\frac{E_{xx}}{E_{zz}}\;;\;\nu_{yz}=\nu_{c}\frac{E_{yy}}{E_{zz}}$$

where $\nu_{c}$ is the Poissons’ ratio of a cell wall.

The shear moduli are calculated as follows:

(4) $$G_{xy}=E_{c}{\left(\frac{t}{l}\right)}^{3}\frac{1+\sin\theta }{3\cos\theta }\;;\;G_{xz}=G_{yz}=G_{c}\left(\frac{t}{l}\right)\frac{\cos\theta }{1+\sin\theta }$$

where $G_{c}$ is shear modulus of the cell wall

#### 2.3.2. Extruded Core

The section below focuses on describing mathematical equations for obtaining homogenized properties of the cell produced using extruded core manufacturing processes.

**Figure 6:**
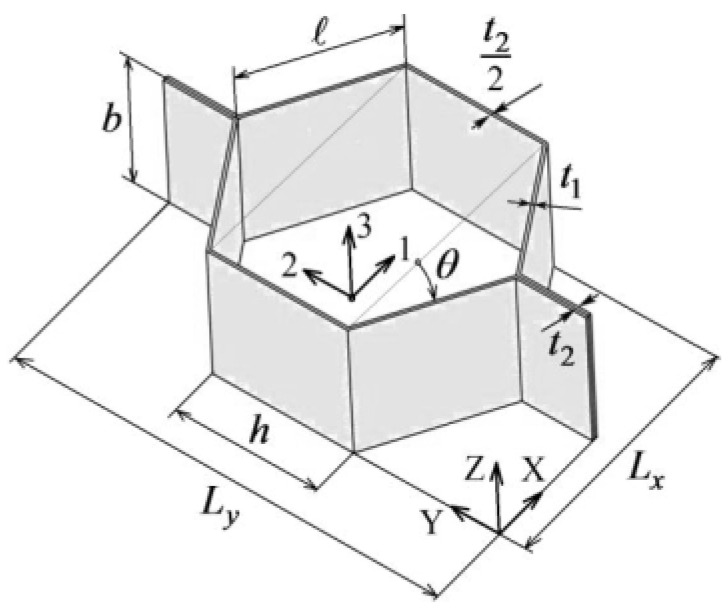
Honeycomb core cell with equation symbols listed for extruded core.

Figure 6, below, is used as a reference for symbols used in following Equations (1)–(13).

Equations allowing us to calculate properties of the honeycomb core produced in the extrusion method have been proposed by S. Sorohan et al. [20].

In the case of this production method, the relative density can be calculated as in (1).

Youngs’ moduli in X, Y, and Z directions:

(5) $$E_{xx}=k_{1}E_{s}{\left(\frac{t}{l}\right)}^{3}\frac{\cos\theta }{\left(\frac{h}{l}+\sin\theta \right)\sin^{2}\theta };\;E_{yy}=k_{2}E_{s}{\left(\frac{t}{l}\right)}^{3}\frac{\frac{h}{l}+\sin\theta }{\cos^{3}\theta }{;\;E}_{zz}=E_{s}\frac{\rho }{\rho_{s}}$$

where

(6) k1=11+tl22.4+1.5νs+cot2θ ;  k2=11+tl22.4+1.5νs+tan2θ+2hl cos2θ

The coefficients, or correction factors, $k_{1}$ and $k_{2}$ become equal to 1 if the axial and shearing force contributions to the total strain energy are neglected [20].

Poissons’ ratios:

(7) $$\nu_{xy}=c_{1}\frac{{cos}^{2}\theta }{\left(\frac{h}{l}+\sin\theta \right)\sin\theta };\;\nu_{yx}=\frac{c_{1}c_{2}}{\nu_{xy}}$$

(8) $$\nu_{zx}=\nu_{zy}=\nu_{s};\;\nu_{xy}=\nu_{s}\frac{E_{xx}}{E_{zz}};\;\nu_{yz}=\nu_{s}\frac{E_{yy}}{E_{zz}}$$

where

(9) $$c_{1}=\frac{1+{\left(\frac{t}{l}\right)}^{2}\left(1.4+1.5\nu_{s}\right)}{1+{\left(\frac{t}{l}\right)}^{2}\left(2.4+1.5\nu_{s}+{cot}^{2}\theta \right)}$$

(10) c2=1+tl21.4+1.5νs1+tl22.4+1.5νs+tan2θ+2hl cos2θ

Shear moduli in XY and XZ planes:

(11) $$G_{xy}=G_{s}{\left(\frac{t}{l}\right)}^{3}\frac{\frac{h}{l}+\sin\theta }{{\left(\frac{h}{l}\right)}^{2}\left(1+\frac{h}{4l}\right)\cos\theta }\;;\;G_{xz}=G_{s}\left(\frac{t}{l}\right)\frac{\cos\theta }{\left(\frac{h}{l}+\sin\theta \right)}$$

Shear moduli in the YZ plane are calculated as upper and lower bounds:

(12) $$G_{s}\left(\frac{t}{l}\right)\frac{\frac{h}{l}+\sin\theta }{\left(\frac{h}{l}+1\right)\cos\theta }\leq G_{xy}\leq G_{s}\left(\frac{t}{l}\right)\frac{\frac{h}{l}+\sin^{2}\theta }{\left(\frac{h}{l}+\sin\theta \right)\cos\theta }$$

The coefficients, or correction factors, $c_{1}$ and $c_{2}$ become equal to 1 if the axial and shearing force contributions to the total strain energy are neglected [20].

## 3. Results

### 3.1. Analytical Calculation and Comparison with Commercial Software Output

For demonstration purposes, the activity of determining the orthotropic properties of extruded honeycomb in the plane (Z) direction using analytical calculation is conducted and used for comparison with ANSYS Material Designer^®^ output.

In this study, ANSYS Material Designer^®^, an application that enables us to model and analyze microstructures and derive homogenized material properties, is utilized in the FEM part to obtain homogenized material properties of the honeycomb core. However, it is important to note that ANSYS Material Designer functions as a “black box,” meaning its internal algorithms and processes are not accessible to the end user; as a result, we cannot rely solely on this software for our analysis.

To address this, we have derived analytical relationships to determine the orthotropic properties of the honeycomb structure. These relationships are then compared with the results obtained from ANSYS Material Designer^®^ for various input parameters. This comparison allows us to validate our analytical approach and understand the limitations and capabilities of the software.

Based on the derived relationships and the comparison of results, we propose a set of guidelines for using ANSYS Material Designer^®^ for homogenization purposes. These guidelines are intended to help users effectively utilize the software while being aware of its limitations.

By following these guidelines, users can effectively utilize ANSYS Material Designer^®^ for homogenization purposes while maintaining a critical perspective on its results. This approach ensures a comprehensive understanding of the material properties and enhances the reliability of the analysis.

The material used as a base for the core structure for this investigation is Aluminum alloy 6061; its given properties were collected from a data sheet:

$\rho_{s}$—= 2770 kg/m^3^,

$E_{s}$— = 71 GPa,

$\nu_{s}$— = 0.33,

$G_{s}$ = 26.7 GPa.

The investigated core dimensions are (refer to Figure 6):

b = 18 mm,

$t_{1}$ = t_2_ = 0.07 mm,

h = 3.66 mm,

l = 3.66 mm,

*θ* = 30 deg,

$L_{x}$, = 6.55 mm,

$L_{y}$, = 11.1 mm.

Using Equations (1)–(12), the calculated material properties for the above-described core are listed in Table 1 and compared with Material Designer output for the same geometry of the cell.

The general comparison between the two approaches is quite accurate; the only values that stand out as higher for analytical calculation (still in the same magnitude order) is in plane shear modulus Gxy and cross-section shear modulus Gyz. The values of the shear moduli Gxz obtained in both methods are in very good agreement.

This points to the problem of blindly trusting the software in terms of the results, which is often the case nowadays. Verification and checks against testing or analytical calculation need to remain present. Understanding the range of applicability and potential sources of error allows for a more informed interpretation of the results and helps in identifying areas where further investigation may be needed.

By assuming the addition of 1 mm face-sheets to the 20 mm core considered above, equivalence in plane mechanical properties can be obtained for a layered stack of those components using the Mixture Rule [19].

The elastic modulus of the layered body is calculated as follows:

(13) $$E_{c}=E_{xx}V_{xx}+E_{yy}V_{yy}$$

The calculated result of the elastic modulus $E_{c}$ was 7720 MPa, which corresponds quite accurately to 7682.2 MPa as obtained from the Material Combination feature in ANSYS (difference c.a. 0.5%).

Other mechanical properties of the layered panel can be estimated in a similar manner, knowing the volume relation between layers.

Alternatively, and more accurately, one can utilize the Laminate Analysis Method, which combines the stiffness matrix of each layer to create a laminate stiffness matrix [19].

### 3.2. Some Guidelines for Using ANSYS Material Designer^®^

It is imperative to clearly define and comprehend the input parameters required by ANSYS Material Designer^®^. These parameters encompass material properties, geometric dimensions, and boundary conditions. A thorough understanding of these inputs is essential for accurate and reliable results.

To ensure the accuracy of the results obtained from ANSYS Material Designer^®^, it is crucial to validate them using analytical methods. This practice helps in identifying any discrepancies and enhances the credibility of the software’s output.

Conducting sensitivity analysis by varying the input parameters is vital to understand their impact on the homogenized properties. This analysis provides valuable insights into the robustness and reliability of the results, allowing for a more comprehensive evaluation of the material properties.

Detailed documentation of all input parameters, assumptions, and results is essential for the transparency and reproducibility of the analysis. Proper reporting ensures that the methodology and findings can be reviewed and replicated by other researchers, thereby contributing to the scientific rigor of the study.

### 3.3. FE Modeling

The equivalent model for a single honeycomb core panel for ANSYS simulation development is described in this section.

There are certain assumptions that must be met for homogenization to be possible [19]:

- Honeycomb structures are periodic,
- Cell structure is uniform and perfect,
- Honeycomb structures are made of one orthotropic linear-elastic material,
- Bonding between cells is perfect.

**Figure 7:**
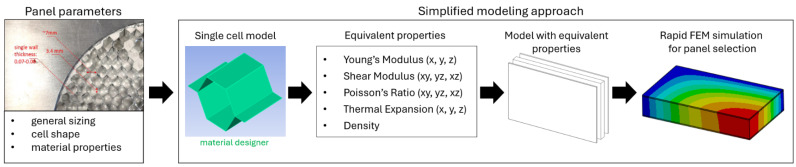
Process flow of the core simplification and analysis.

The process map of homogenization and analysis of the honeycomb panels is presented in Figure 7. It starts with the panel’s geometric and material properties, and goes through simplification based on single cell modeling to final FEM analysis.

### 3.4. Levels of Complexity

**Figure 8:**
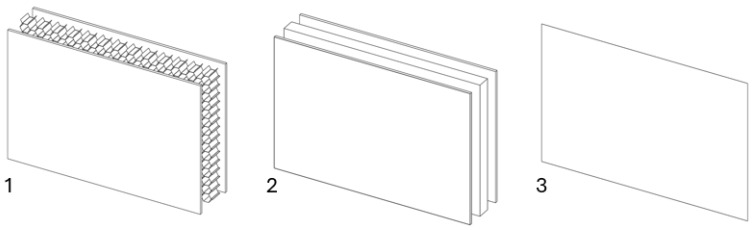
Visualization of three levels of honeycomb modeling complexity.

When modeling honeycomb cores, one can use various levels of computational complexity; three possibilities are listed below (visible in Figure 8):

1. Based on the full shapes of the core and skins (heaviest, time consuming, most accurate).
2. Based on orthotropic material properties using homogeneous bodies—solids (easy to model, fast, accurate, allows us to extract forces between skins and core).
3. Based on orthotropic material properties using shells (lightest, fastest, potentially less accurate).

All mentioned levels will be presented and investigated in following sections.

#### 3.4.1. Level 1: Fully Modeled Shape (L1)

The initial approach is aimed at modeling the complete structure of the panel, involving detailed representation of each individual feature in the geometry. This approach provides a direct depiction of the stress-and-strain distribution across the structure. However, a notable drawback of this method is the significant computational effort it demands; hence, a long time is required to obtain the results.

The modeling task was focused on three kinds of components that each standard honeycomb composite panel consists of:

1. Skins (face-sheets)
  The simplest components to define, as they can be represented either by a single shell or a solid block, depending on the chosen modeling approach (here, a full solid model was created).
  When considering skins, only two variables need to be taken into account: thickness and material type (size matches the overall panel size). It is naturally possible to have different thicknesses and material types for each side of the panel, if necessary.
2. Bonding adhesive
  Regarding the adhesive, the chosen approach involved defining the contact within the finite element model as bonded and evaluating the shear stress transmitted through it during post-processing.
  If such evaluated shear stress exceeds the allowable limit determined through tests on the components, it would indicate that the bonding fails to meet the required criteria.
3. Honeycomb core
  The honeycomb core in this modeling approach is fully modeled. Each single core cell is patterned in the Y and Z direction to match the required shape (excess is cut off).

#### 3.4.2. Level 2: Solids Using Homogenization (L2)

Further investigation led to simplification of the honeycomb structure in the form of a homogeneous block with equivalent orthotropic material properties, according to the method described in Section 3, Core Homogenization.

Results of the homogenization were implemented as orthotropic material properties for the core, which was modeled as a solid block. The Level 2 approach boiled down to the creation of a structure consisting of three solid layers (skin–core–skin) and the application of the obtained orthotropic properties to the middle body.

#### 3.4.3. Level 3: Shells Using Homogenization (L3)

The final step of simplification was the shell approach. Computation-wise, it should be the fastest approach. For achieving this goal, a shell model was considered using a multi-layer shell element with quadratic formulation.

Using the classical laminate theory, stiffness properties were determined for a three-layer element, for which the outer layers had the properties of aluminum, and the middle layer was determined from equations based on the adopted homogenization process to obtain appropriate orthotropic properties.

Using this method, a single shell is created and material properties are specified for each artificially created layer [19].

### 3.5. Mesh Convergence Study

To verify Material Designer against the analytical approach and to check which accuracy of meshing proves to be sufficient and most efficient against limited calculation resources, a mesh convergence study was performed with both hexahedral and tetrahedral mesh types.

#### 3.5.1. Hexahedral Mesh Convergence Study

In the following tables, one can see the results of the mesh study set against the analytical calculation (Table 2) for increasingly dense hexahedral mesh ranging from 1 mm to 0.1 mm in size. In all cases, element formulation was quadratic.

**Figure 9:**
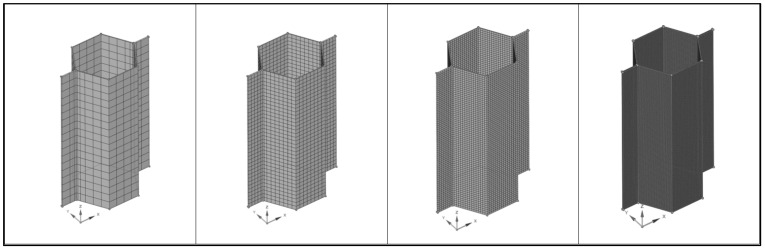
Mesh convergence study cell visualization for hexahedral mesh—from left to right: 1; 0.5; 0.25; 0.1 mm.

In Figure 9, mesh visualizations for each considered case is shown.

**Figure 10:**
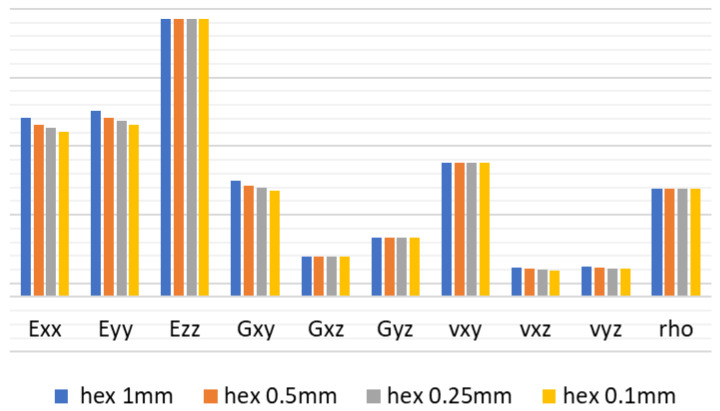
Hexahedral mesh convergence study results trend.

As shown in Figure 10, decreasing the size of the mesh provided a significant accuracy increase in comparison with analytical calculation.

#### 3.5.2. Tetrahedral Mesh Convergence Study

**Figure 11:**
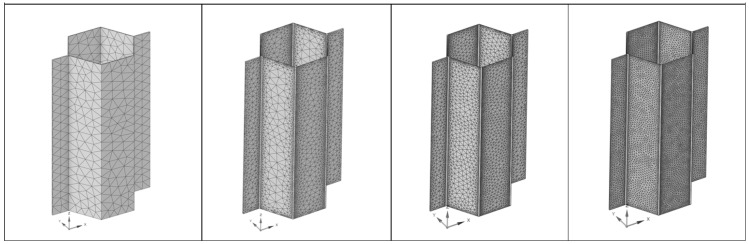
Mesh convergence study cell visualization for tetrahedral mesh—from left to right: 1; 0.5 mm standard distribution and 1; 0.5; 0.25 mm adaptive edge distribution.

In the following table and Figure 11, one can see the results of the mesh study set against the analytical calculation (Table 3) for increasingly dense tetrahedral meshes for 1 mm and 0.5 mm sizes with standard setting, and for 1 mm, 0.5 mm, and 0.25 mm with adaptive mesh, which provides much finer results close to the edges. In all cases, element formulation was quadratic.

**Figure 12:**
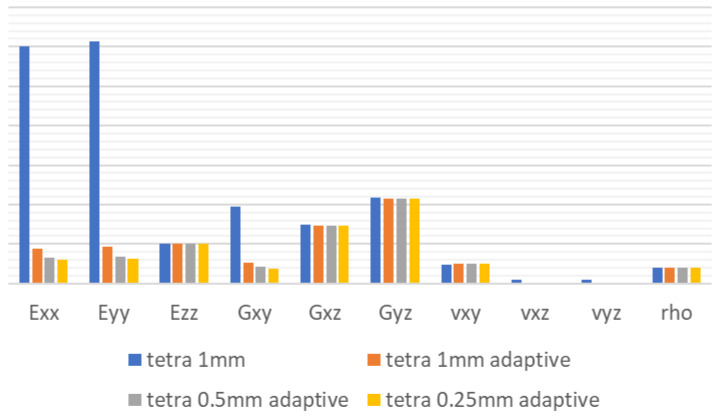
Tetrahedral mesh convergence study results trend.

As shown in Figure 12, the standard mesh setting for the tetrahedral shape is not usable in this case due to over-stiffening of the model, which leads to unrealistic results. Adaptive mesh settings close to the edges eliminate that effect and provide similar behavior to previously shown hexahedral mesh—decreasing the size of the mesh provided an accuracy increase in comparison with analytical calculation.

Observations from the mesh convergence studies are listed below.

Results shown in Table 2 and Table 3 led to following observations:

1. Analytical vs. Material Designer calculations for the orthotropic properties of a honeycomb core show significant differences.
2. Differences are also visible between Material Designer calculation itself, depending on the mesh type selected and refinement level.
3. Hexahedral mesh proves more accurate than tetrahedral mesh in comparison to analytical calculations on lower mesh quality levels; however, the difference is non-existent with higher refinement levels.
4. Shear in the XY plane (Gxy) provides the highest differences between analytical and material designer calculations, which are significant even after mesh refinement.
5. Tetrahedral mesh tends to stiffen the structure significantly when coarse and without adaptive edge setting.
6. On the highest level of refinement checked, there is practically no difference in the results between tetrahedral and hexahedral mesh.

Further analysis was based on hexahedral mesh.

### 3.6. Boundary Conditions and Its Effect on Results

There is a significant difference in the definition of the constraining boundary conditions of shell and solid bodies. A different formulation is used by the ANSYS 2019R3 software.

**Figure 13:**
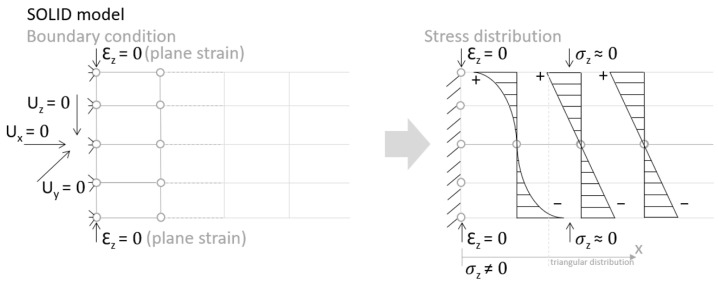
Stress distribution and boundary condition visualization for the solid model.

Let us consider the case of the solid model in which the clamping region is under a plain strain condition. The displacement of the fixed nodes of a solid model is constrained in all directions, and this gives rise to an additional stress in the boundary condition due to the forcing εz = 0 on the clamping region (see Figure 13).

**Figure 14:**
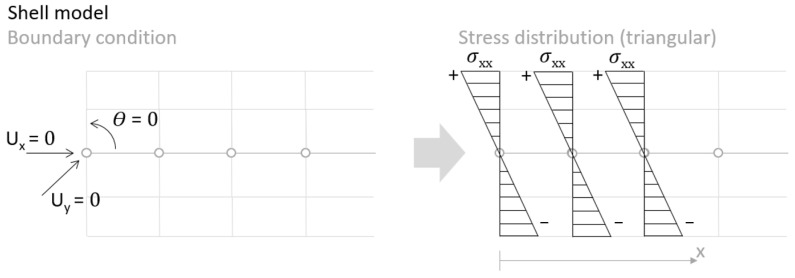
Stress distribution and boundary condition visualization for SHELL model.

Shell formulation is based instead on the plane stress condition, therefore no additional stress component is introduced when we fix a shell rotation (see Figure 14).

**Figure 15:**
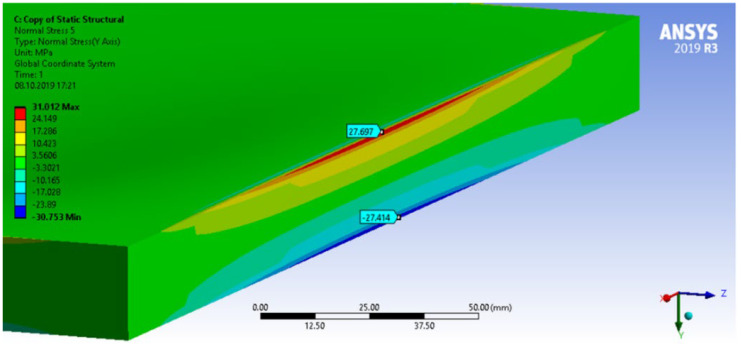
Out-of-plane stress is not zero at the edge, where BC is applied [22].

In case of the solid model, the plane strain boundary condition generates additional out-of-plane stresses on the outermost edges of the clamping region, as seen in the Figure 15 example.

The reason for the triggering of this numerical singularity can then be attributed to the fixed boundary condition on the solid elements, which is forcing a plain strain status for the clamping region.

This generates an additional stress component that is not present in the plain stress condition.

**Figure 16:**
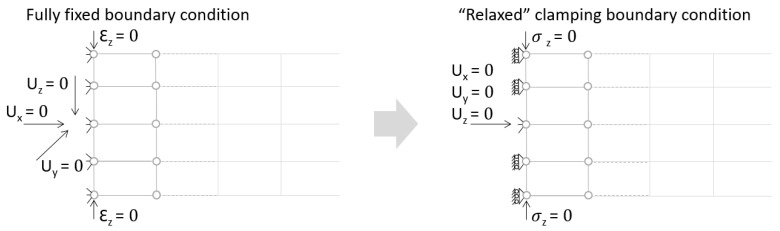
Boundary conditions representation for the solid model.

The proposed solution to the described problem is, as shown in the Figure 16, relaxing the boundary condition in the out-of-plane direction for the solid model. This allows us to eliminate the plane strain problem in the clamping region. Only the mid-plane nodes keep a fixed constraint in order to prevent rigid body motion of the plate. For all other nodes of the clamped section, a “frictionless” support is enough to prevent any motion or rotation of the entire section. No displacement condition on the out-of-plane direction is imposed.

### 3.7. Comparison of Proposed Simplification Methods

The results’ comparisons in the form of tables and figures for the assumed models is presented in this section. General results were obtained on a ¼ model using symmetry planes, whereas modals were performed on the full model (no symmetry planes so as not to omit nonsymmetric modes).

As seen in Table 4, numerical models L1 and L2 provide identical results except for the core analysis singularities near the border—this is due to the accurate representation that L1 provides, and the local peak of stresses. When comparing models L1 and L2 to L3, there is some difference coming from simplification; this is especially prominent in relatively small models like the one used in this study. When using layered cross-sections in L3, values are extrapolated from the single 2D sheet that is in the middle of the sandwich panel; hence, the results in the middle (core) are the same as in L2, but a difference is seen on the face-sheets.

When comparing normal stresses in direction X, all three sets of results are comparable. The Von-Mises model emphasizes boarder conditions of support, where a singularity is present; hence, the difference is more prominent. Lack of symmetry in the L1 model (−25 to 28 MPa) comes from the lack of continuity of the structure in the geometry of the core.

**Figure 17:**
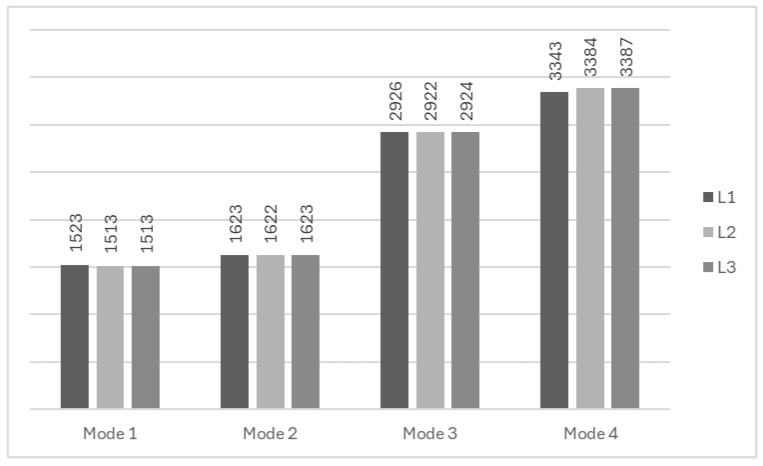
Natural frequency [Hz] comparison between different simplification levels of FEM.

The modal analysis results (see Figure 17 and Table 5) are almost identical for all assumed models. The differences between natural frequencies and the corresponding modes are negligibly small.

### 3.8. Verification

The experimental tests were conducted by the Hitachi Energy (former ABB) company. The results partially quoted here were fully described in reference to two internal reports by L. Michalski and G. Moroni, describing four-point bending testing performed on the honeycomb panels to validate the simulation. Testing was performed according to the ASTM D7249 [22] testing methodology.

**Figure 18:**
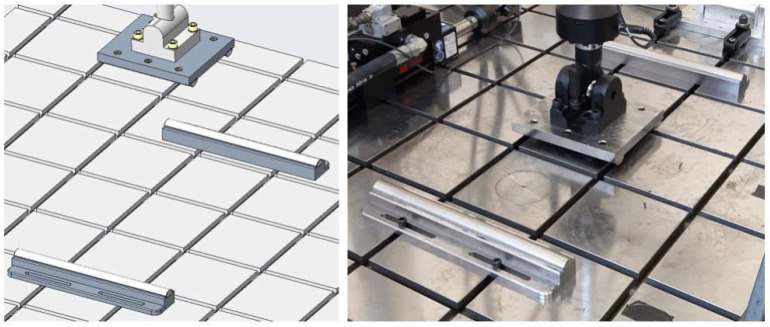
Testing setup—overview of CAD model and actual rig.

The employed setup consists of two cylindrical supports and two cylindrical loading bars, both free to rotate around their main axes, as can be seen in Figure 18.

**Figure 19:**
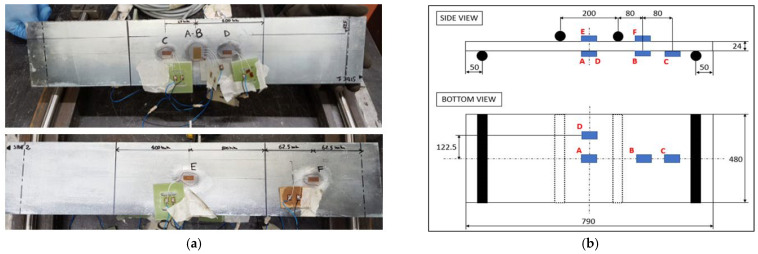
(**a**) picture of tested sample; (**b**) strain gauge positioning during single panel testing.

A sample panel was equipped with strain gauges (LY11-6/120A from HBM system), as it is presented in Figure 19.

Additionally, laser sensor measurements of the displacement in the center of the panel were taken using an Mirco-Epsilon Opto NCDT 1220 (sourced from WObit, Pniewy, Poland) displacement transducer.

### 3.9. Test Results

In parallel to physical testing, an identical setup was recreated in an ANSYS environment. Accurate modelling of the panel, supports, and load was utilized to obtain results which could be compared with and used to confirm real-life testing.

**Figure 20:**
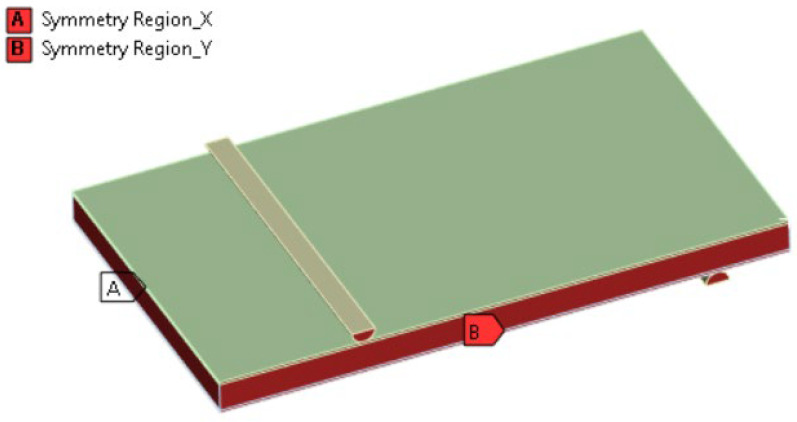
Visualization of considered FEM model.

Several simplifications were introduced to the simulation model of the single panel (Figure 20). Primarily, an introduction of an orthotropic model of the panel (solid approach), as described in earlier chapters regarding simulation development. There were also symmetry-based simplifications introduced for the supports (half of the bars) and the overall model (quarter of the structure).

**Figure 21:**
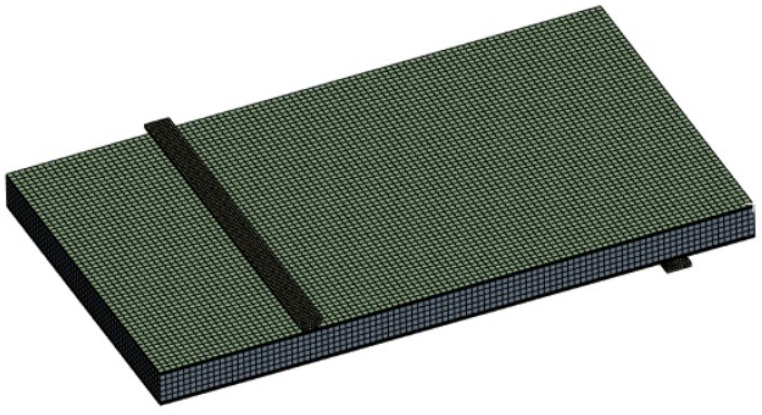
Mesh visualization for considered FEM model.

A quarter of the panel and two halves of the supports are discretized using hexahedral elements, sized in a way that there are at least two elements along the thickness of the skins and the core (Figure 21). The panel surface rollers require nodal inflation near the contact zone, since this is crucial for the non-linear contact formulation that takes place between the two. Coarse mesh in this region can lead to severe simulation problems, up to complete non-convergence.

Additionally, for comparison purposes, analytical calculation for the flexure of the composite beam was performed, as described by Allen H. in [13].

The simulation model is based on Level 2 simplification described previously—solids with homogenized cores. Strain gauge positions referred to can be seen in Figure 19b. Deflection measurement was collected with laser displacement sensors at the center of the panel from the top layer.

**Figure 22:**
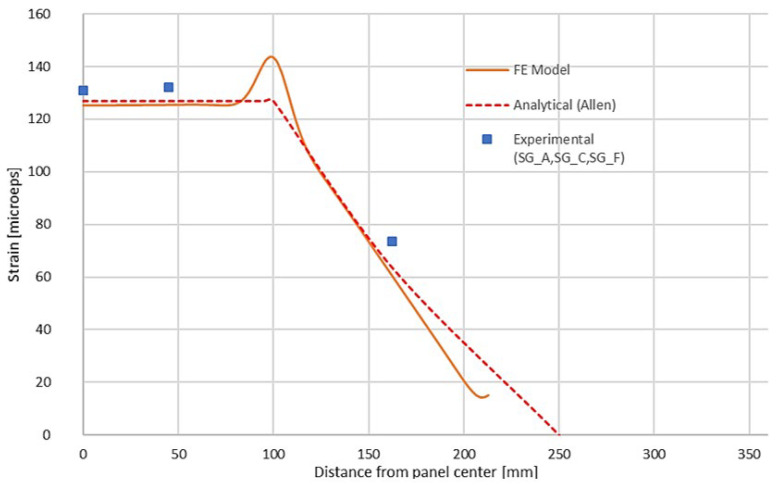
Normal strain along panel centerline—combined graph for three considered cases.

**Figure 23:**
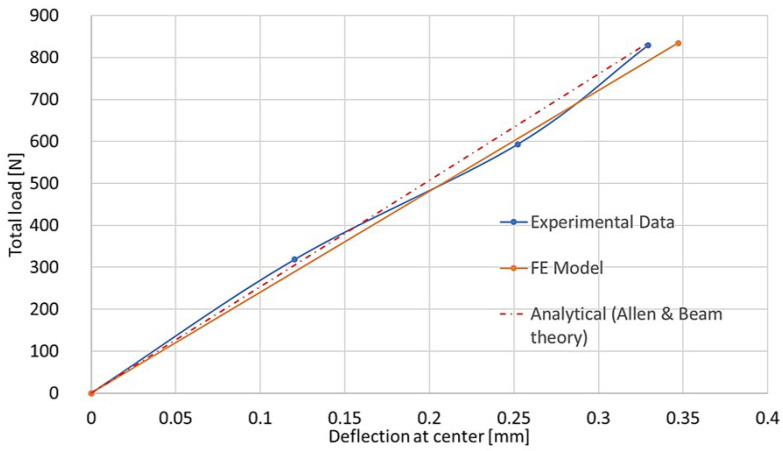
Deflection vs. total load—combined graph for three considered cases.

**Figure 24:**
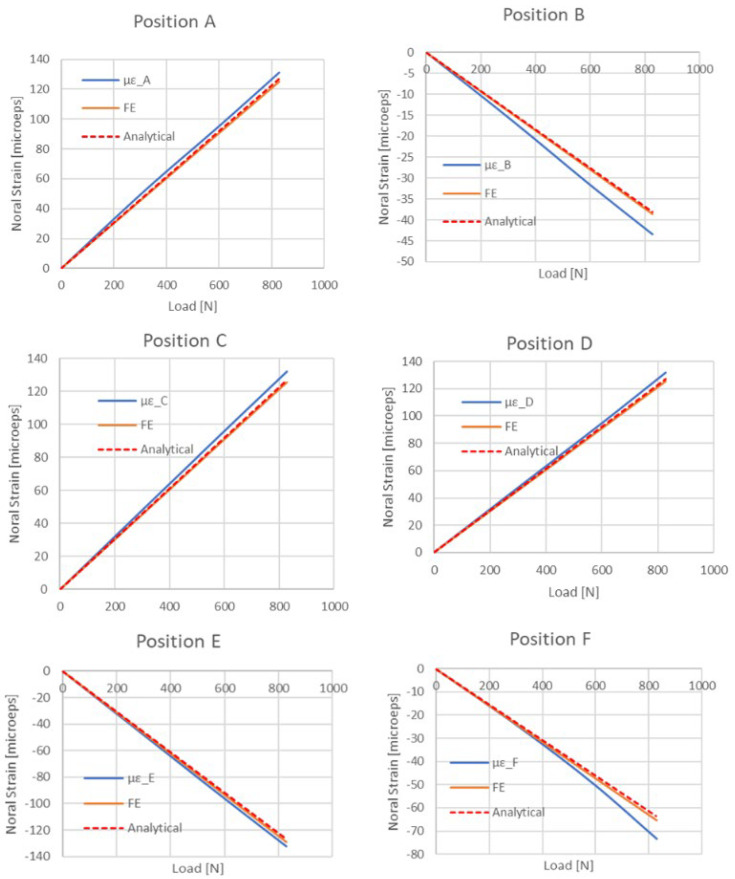
Normal strain vs. load for each SG position—combined graph for three considered cases.

Figure 22, Figure 23 and Figure 24 show the result comparison between the FE model, the experimental data, and analytical calculations.

Good correlation between all three is visible on the load–deflection curve (Figure 23). The normal strain along the panel center (Figure 22) matches well, with significant difference only in the region of support, which is described in a simplified way by the analytical approach. For normal strain vs. load for each SG position (Figure 24), an ideal correlation is present between analytical and FE approaches, whereas experimental results are slightly different—this may be attributed to the quality of the strain gauge application and the impurities of the real structure. Nevertheless, those results are also very much correlated.

## 4. Discussion

The focus of this study was placed on finding and validating an efficient finite element model for honeycomb core composite panels, enabling straightforward and accurate simulation. It emphasizes the use of the homogenization method to derive equivalent orthotropic mechanical properties for the honeycomb core, simplifying the analysis of these complex structures and removing necessity for complex geometry creation. Three finite element modeling approaches, from a fully detailed three-dimensional core to simplified two-dimensional shell models, were investigated in terms of complexity, accuracy, and computational effort.

Representative Volume Element (RVE) simulations were used to calculate equivalent orthotropic properties, ensuring fast and reliable modeling while preserving accuracy.

The proposed FE models were validated through experimental four-point bending tests, analytical calculations, and comparisons with prior research. All three approaches (numerical, analytical, and experimental) demonstrated strong agreement.

A trade-off between accuracy and computational efficiency was explored, with recommendations provided for applications requiring high precision versus rapid iterative simulations.

Simplification levels should be adjusted to the complexity of the investigated models—smaller, less complex models should be kept in accurate representation (L1), and with higher complexity, higher levels of simplification (L2) should be introduced. Depending on the boundary condition application in numerical models, as they are close to real conditions, one can consider using simplification L2 or L3 on a case-to-case basis, having in mind the theory which describes the various types of elements. 

It is necessary to remember to be careful in choosing tool-based auto-simplifications, as was presented with core homogenization and ANSYS Material Designer^®^ usage. One must always be inclined to check and verify the correctness of the results coming from automated tools.

Special attention was given to accurate boundary condition applications and resolving numerical singularities to enhance model reliability. The proposed solution of relaxing the boundary condition in the out-of-plane direction for the solid model eliminated the numerical singularity identified on the edges of the constrained panel.

## 5. Conclusions

The findings contribute to the design and optimization of honeycomb panels in industries such as aerospace, automotive, marine, construction, and beyond.

Best practices for honeycomb sandwich panel modeling drawn as a result of the described study are as follows:

1. Validate the model, either by testing or comparison with analytical calculation.
2. If using tools, e.g., ANSYS Material Designer^®^, clearly define and comprehend the input parameters required: material properties, geometric dimensions, and boundary conditions.
3. For most approaches, the best method of modeling will be through the use of orthotropic material properties on homogeneous bodies—solids (easy to model, fast, accurate, allows us to extract forces between skins and core).
4. Sufficiently small mesh of either hexahedral or tetrahedral elements with quadratic formulation will provide accurate results.
5. For analysis, use “relaxed” boundary conditions (refer to chapter 5 for details) or try to set them as close as possible to the real ones.

## Figures and Tables

**Table 1:** Comparison of analytical calculation and Material Designer homogenization results for 0.1 mm quadratic hex element expanded core.

	Unit	Analytical Calculation	Material Designer	Difference [%]
Exx	MPa	1.14	1.202	−4.8%
Eyy	MPa	1.15	1.253	−8.6%
Ezz	MPa	2045	2024	1.0%
Gxy	MPa	0.344	0.773	−55.5%
Gxz	MPa	295	294	0.2%
Gyz	MPa	295	430	−31.4%
vxy	-	0.999	0.978	2.2%
vxz	-	0.0002	0.0002	0%
vyz	-	0.0002	0.0002	0%
rho	kg m^−3^	79.77	78.96	1.0%

**Table 2:** Hexahedral mesh convergence study results set against analytical calculation.

	Unit	Analytical	Hex 1 mm		Hex 0.5 mm		0.25 mm		Hex 0.1 mm	
Exx	MPa	1.14	1.31	14%	1.26	10%	1.23	8%	1.20	5%
Eyy	MPa	1.15	1.36	19%	1.31	14%	1.28	12%	1.25	9%
Ezz	MPa	2045	2024	−1%	2024	−1%	2024	−1%	2024	−1%
Gxy	MPa	0.34	0.85	146%	0.81	135%	0.79	131%	0.77	125%
Gxz	MPa	295	294	0%	294	0%	294	0%	294	0%
Gyz	MPa	295	430	46%	430	46%	430	46%	430	46%
vxy	-	0.9993	0.9783	−2%	0.9781	−2%	0.9781	−2%	0.9781	−2%
vxz	-	0.0002	0.0002	15%	0.0002	11%	0.0002	8%	0.0002	6%
vyz	-	0.0002	0.0002	20%	0.0002	15%	0.0002	13%	0.0002	10%
rho	kg m^−3^	79.77	78.96	−1%	78.96	−1%	78.96	−1%	78.96	−1%

**Table 3:** Tetrahedral mesh convergence study results set against analytical calculation.

	Unit	Analytical	Tetra 1 mm		Tetra 1 mm Adaptive		Tetra 0.5 mm Adaptive		Tetra 0.25 mm Adaptive	
Exx	MPa	1.14	12.00	948%	1.79	56%	1.32	16%	1.19	4%
Eyy	MPa	1.15	12.29	973%	1.86	63%	1.38	21%	1.25	9%
Ezz	MPa	2045	2024	−1%	2024	−1%	2024	−1%	2024	−1%
Gxy	MPa	0.34	3.90	1033%	1.05	206%	0.84	143%	0.77	124%
Gxz	MPa	295	298	1%	294	0%	294	0%	294	0%
Gyz	MPa	295	437	48%	430	46%	429	46%	429	46%
vxy	-	0.9993	0.9752	−2%	0.9778	−2%	0.9780	−2%	0.9780	−2%
vxz	-	0.0002	0.0020	957%	0.0003	58%	0.0002	17%	0.0002	5%
vyz	-	0.0002	0.0020	983%	0.0003	64%	0.0002	22%	0.0002	10%
rho	kg m^−3^	79.77	78.96	−1%	78.96	−1%	78.96	−1%	78.96	−1%

**Table 4:** General comparison of the results between different levels of FEM (1–3).

L1	L2	L3
Computing time		
48 s	22 s (54% decrease)	9 s (81% decrease)
Deflection distribution		
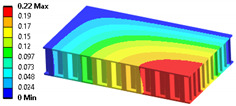	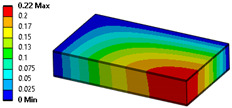	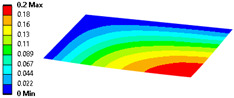
Uz_max_ = 0.22 mm	Uz_max_ = 0.22	Uz_max_ = 0.20
Stress (von-Mises) −Core		
σ_max_ = 257	σ_max_ = 5.6	σ_max_ = 5.3
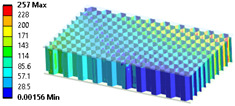	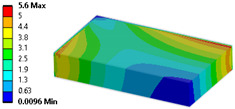	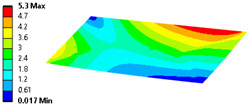
Stress (von-Mises) −Face-sheets		
σ_max_ = 162	σ_max_ = 169	σ_max_ = 52
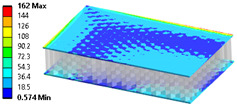	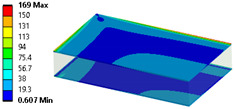	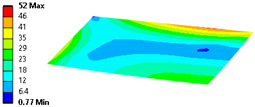
Stress—Normal X—Path through the middle		
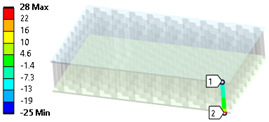	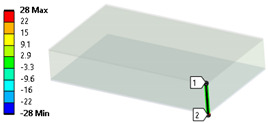	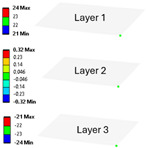
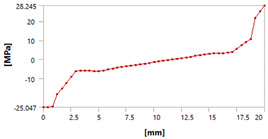	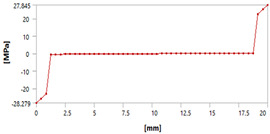	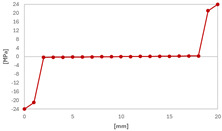

**Table 5:** Comparison of the modal shapes between different simplification levels of FEM.

L1	L2	L3
Mode 1		
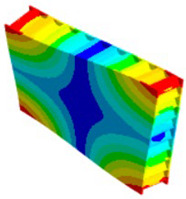	* 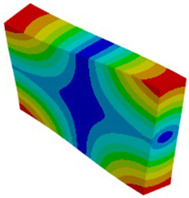 *	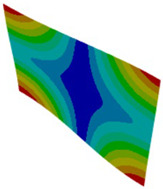
Mode 2		
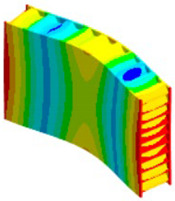	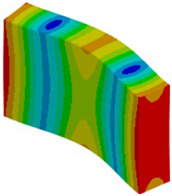	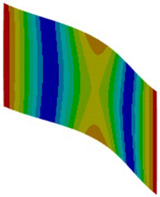
Mode 3		
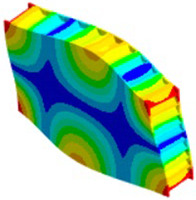	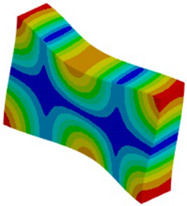	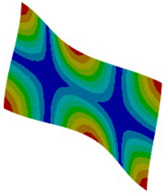
Mode 4		
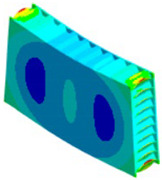	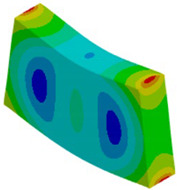	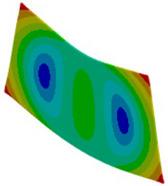

## Data Availability

The original contributions presented in the study are included in the article, and further inquiries can be directed to the corresponding author.
